# Beating the clock: comparing the speed of anticoagulation reversal in intracerebral haemorrhage to thrombolysis for acute ischaemic stroke

**DOI:** 10.1093/esj/aakag011

**Published:** 2026-03-14

**Authors:** Maud B R C Eurlings, Nabila Wali, Maritta N van Stigt, M Irem Baharoglu, Jonathan M Coutinho

**Affiliations:** Department of Neurology, Amsterdam UMC location University of Amsterdam, Meibergdreef 9, Amsterdam 1105 AZ, The Netherlands; Department of Neurology, Amsterdam UMC location University of Amsterdam, Meibergdreef 9, Amsterdam 1105 AZ, The Netherlands; Department of Neurology, Amsterdam UMC location University of Amsterdam, Meibergdreef 9, Amsterdam 1105 AZ, The Netherlands; Department of Neurology, Haaglanden Medical Center, Lijnbaan 32, Den Haag 2512 VA, The Netherlands; Department of Neurology, Amsterdam UMC location University of Amsterdam, Meibergdreef 9, Amsterdam 1105 AZ, The Netherlands

**Keywords:** acute ischaemic stroke, anticoagulant reversal, door-to-needle time, ICH, workflow

## Abstract

**Introduction:**

Intravenous thrombolysis (IVT) for acute ischaemic stroke (AIS) and anticoagulation reversal for intracerebral haemorrhage (ICH) are both emergency stroke treatments. We hypothesized that, given the similar logistics, door-to-needle times should be comparable for both treatments.

**Patients and methods:**

We used data from 2023 and 2024 from the Dutch Acute Stroke Audit, a national stroke registry with 66 contributing hospitals in the Netherlands. We compared door-to-needle time of patients with anticoagulation-related ICH who received a reversal agent to patients with AIS who received IVT.

**Results:**

Of 1,897 adult patients with anticoagulation related ICH, 1018 (54%) received a reversal agent, of whom 579 (57%) had available door-to-needle times. Of 67,699 AIS patients, 14,192 (21%) received IVT and 13,752 (97%) had available door-to-needle times. ICH patients were older (80 years vs 74 years, *P* < .001) and had a higher NIHSS at presentation (7 vs 4, *P* < .001) compared to AIS patients. Onset-to-door times were longer for ICH patients than AIS patients (78 min vs 73 min, *P* < .001). Median door-to-needle time was 49 (IQR: 30–90) min for ICH patients and 28 (IQR: 20–41) min for AIS patients (adjusted β = −0.573; 95% CI, −0.631 to −0.515; *P* < .001).

**Discussion and conclusion:**

In this nationwide study, door-to-needle time was substantially longer for anticoagulation reversal in ICH patients than IVT in AIS patients. This indicates that there is room for improvement in the emergency workflow of ICH patients.

## Graphical Abstract

**Graphical Abstract:**
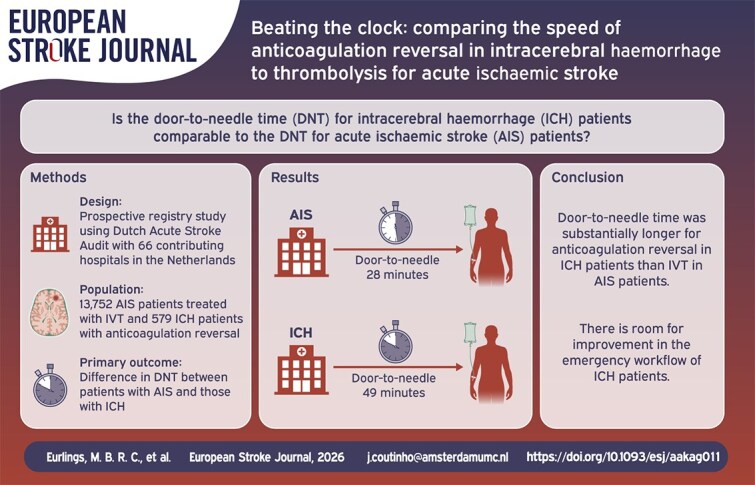

## Introduction

Intracerebral haemorrhage (ICH) is the most devastating subtype of stroke and often leads to severe disability or death.^1^ Anticoagulation use is both a risk factor for developing ICH, as well as a predictor of poor outcome.^2,3^ The risk of ICH is increased approximately 2- to 7-fold in patients using anticoagulants, depending on the type of agent- and patient-related factors.^4,5^ Due to an increase in the incidence of atrial fibrillation, with anticoagulation as standard treatment, the frequency of anticoagulation-related ICH is expected to increase.^6,7^

Anticoagulation-associated ICH has been linked to larger ICH volumes, an increased risk of haematoma expansion and worse functional outcomes, compared to spontaneous ICH.^8–10^ Treatment strategies for ICH are evolving rapidly.^11,12^ Standardized protocols for intensive blood pressure management have been established, surgical interventions are being explored in selected cases and specific reversal agents for anticoagulants are increasingly implemented.^13,14^ The administration of anticoagulation reversal agents reduces the risk of haematoma expansion, as illustrated by data from vitamin K-related ICH, where rates fall from 41.5% without reversal to 19.8% when the international normalized ratio (INR) is corrected to < 1.3.^15^ The ANNEXA-I study showed that andexanet alpha reduces the risk of haematoma expansion in patients with ICH who use a factor Xa inhibitor.^16^ However, the effectiveness of reversal agents is highly time-dependent. Previous studies have demonstrated that INR correction within 4 h after hospital arrival is associated with reduced haematoma enlargement, whereas administration of a reversal agent within one hour has been linked to improved survival.^15,17^ As such, the time = brain paradigm, a deeply engraved concept in acute ischaemic stroke (AIS) treatment, likely also applies to ICH.^18^

The logistics of intravenous thrombolysis (IVT) are similar to that of anticoagulation reversal, and thus similar door-to-needle times (DNTs) should be achievable for both treatments. The aim of the current study was to assess DNT for anticoagulation reversal in ICH patients in the Netherlands, and compare these to the DNT for AIS patients.

## 1. Methods

### 1.1. Study design and population

We used data from the Dutch Acute Stroke Audit (DASA). The DASA is a prospective national stroke registry that includes data from 66 of the 69 hospitals in the Netherlands that provide acute neurologic care, with the aim to monitor and improve the quality of care of stroke patients.^19^

We included all adult patients admitted between 1st January 2023 and 31st December 2024 with either an anticoagulation-associated ICH who received a reversal agent or AIS who were treated with IVT. Patients for whom data on DNT were missing were excluded.

### 1.2. Definitions and outcome

All clinical classifications and medication categories were based on predefined criteria used within the DASA. Ischaemic stroke was defined as the presence of neurological symptoms attributable to the occlusion of a cerebral artery. Patients with transient ischaemic attack (TIA) and infarctions resulting from cerebral venous sinus thrombosis were excluded. ICH was defined as a spontaneous bleeding event within the brain parenchyma. This category excluded primary subarachnoid haemorrhage, subdural haematoma, epidural haematoma, haemorrhage due to arteriovenous malformation and haemorrhagic transformation of space-occupying cerebral lesions. The following medications were classified as anticoagulants: vitamin K antagonists, direct oral anticoagulants (DOACs) and therapeutic heparin. Reversal agents recorded in DASA included prothrombin complex concentrate, DOAC-specific antidotes, protamine and vitamin K.

### 1.3. Data analysis

The primary outcome was the difference in DNT between patients with AIS and those with ICH. Door-to-needle time was defined as the time from hospital arrival to the administration of treatment (ie, anticoagulation reversal for ICH patients and IVT for AIS patients). Secondary outcomes were the mRS scores and mortality at 3 months. We compared baseline characteristics, clinical outcome and DNT between patients with ICH and AIS using the independent samples *t*-test for normally distributed continuous variables, the Mann–Whitney *U* test for non-normally distributed continuous or ordinal variables and the Pearson’s chi-square test for nominal variables. A *P*-value ≤ .05 was considered statistically significant.

Linear regression was performed to further analyse the association between patient diagnosis and DNT. Door-to-needle time was log-transformed to achieve normality of the residuals. Unadjusted beta estimates were obtained using a bootstrap procedure with 5000 iterations. The analysis was adjusted for sex, age and NIHSS. Missing data were handled using listwise deletion for the regression analyses, whereby cases with missing values on any of the included variables were excluded from the analysis. To assess the robustness of the results on DNT, mortality and mRS 0–2 with respect to missing data, a sensitivity analysis using multiple imputation was performed in the total group AIS patients treated with IVT and ICH patients treated with a reversal agent following Rubin’s Rules for multiple imputation pooling.^20,21^ Missing values in mRS and DNT were imputed using multiple imputation. Age, sex, diagnosis and NIHSS were included in the imputation model as predictors.

An additional sub-analysis was performed to compare baseline characteristics for ICH patients treated with a reversal agent for whom DNT was available with those for whom this information was not available. A second subgroup analysis was performed to compare baseline characteristics, including the number and proportion of patients receiving a reversal agent, of anticoagulated ICH patients with known vs unknown time of onset.

## 2. Results

**Figure 1:**
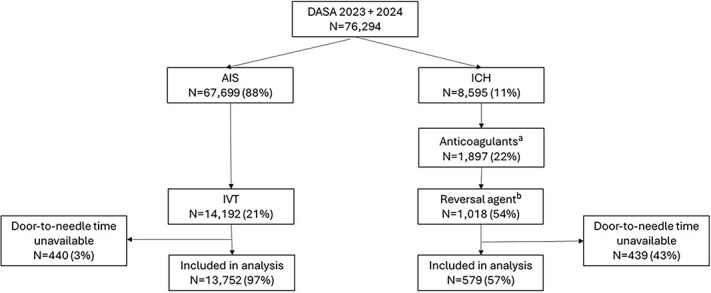
Patient flowchart. Abbreviations: DASA = Dutch Acute Stroke Audit; AIS = acute ischaemic stroke; ICH = intracerebral haemorrhage; IVT = intravenous thrombolysis. Number of missing values: ^a^2,415; ^b^79.

In 2023 and 2024, data of 76,294 stroke patients were included in the DASA registry. Of these, 67,699 (88%) had AIS and 8,595 (11%) had ICH. Of the patients with AIS, 14,192 (21%) were treated with IVT. After excluding patients with missing DNT (*n* = 440), data of 13,752 AIS patients were included in the analysis. Of the ICH patients, 1,897 (22%) used anticoagulation at presentation, and in 1,018/1,897 patients (54%) anticoagulation was reversed using a reversal agent. We excluded 439 ICH patients due to unavailable DNT, resulting in 579 ICH patients included in the analysis (Figure 1).

ICH patients were older (median 80 years vs 74 years, *P* < .001) and had higher baseline NIHSS scores (7 vs 4, *P* < .001; Table 1) than patients with AIS. There was no significant difference in sex distribution. The onset-to-door time was longer for ICH patients than AIS patients (78 min vs 73 min, *P* < .001), and a smaller proportion of ICH patients had a known time of onset (53.5% vs 83.3%, *P* < .001).

**Table 1:** Baseline characteristics.

	**ICH (*n* = 579)**	**AIS (*n* = 13,752)**	***P*-value**
Age, years—median (IQR)	80 (74–85)	74 (63–81)	<.001
Sex, male—*n/N* (%)	348/569 (61.2%)^a^	7,473/13,233 (56.5%)^b^	.027
NIHSS—median (IQR)	7 (3–13)^c^	4 (2–8)^d^	<.001
Onset-to-door time, minutes—median (IQR)	78 (51–227)^e^	73 (49–122)^f^	<.001
Known time of onset—*n/N* (%)	310/579 (53.5%)	11,465/13,752 (83.3%)	<.001

Abbreviation: AIS = acute ischaemic stroke; ICH = intracerebral haemorrhage.

Number of missing values: ^a^10; ^b^519; ^c^110; ^d^1,437; ^e^272; ^f^2,352.

ICH patients had a median DNT of 49 min, compared to 28 min for AIS patients (adjusted β [95% CI]: −0.573 [−0.631 to −0.515], *P* < .001; Table 2). Clinical outcomes were worse for ICH patients, with a lower proportion achieving a favourable outcome (mRS 0–2) (24.0% vs 69.4%, *P* < .001; Table 2), and higher mortality at 3 months (50.4% vs 13.9%, *P* < .001; Table 2). In the sensitivity analysis using multiple imputation, we still found a statistically significant association with DNT (unadjusted β [95% CI]: −0.537 [−0.585 to −0.489], adjusted β [95% CI]: −0.585 [−0.642 to −0.528]). The difference in mRS and mortality also remained statistically significant (both *P* < .001).

**Table 2:** Door-to-needle time and clinical outcome.

	**ICH (*N* = 579)**	**AIS (*N* = 13,752)**	***P*-value**	**Unadjusted β (95% CI)**	**Adjusted β (95% CI)** ^*^
Door-to-needle time, minutes—median (IQR)	49 (30–90)	28 (20–41)	<.001	−0.535 (−0.607 to −0.460)^a^	−0.573 (−0.631 to −0.515)^b^
mRS 0–2 at 3 months follow-up—*n/N* (%)	117/488 (24.0%)^c^	6,929/9,989 (69.4%)^d^	<.001	Not applicable	Not applicable
Mortality at 3 months—*n/N* (%)	246/488 (50.4%)^c^	1,391/9,989 (13.9%)^d^	<.001	Not applicable	Not applicable

Abbreviation: AIS = acute ischaemic stroke; ICH = intracerebral haemorrhage.

Number of missing values: ^a^7; ^b^20,309; ^c^91; ^d^3,763.

^*^Adjusted for age, sex, and NIHSS.

When comparing patients with ICH with available and missing DNT, patients with available DNT were older (80 years vs 78 years, *P* = .006; Table 3). There were no differences in baseline NIHSS, onset-to-door time or 3-month mRS and mortality.

**Table 3:** Sub-analysis ICH patients treated with a reversal agent, stratified by availability of door-to-needle time.

	**Available DNT (*n* = 579)**	**Unavailable DNT (*n* = 439)**	***P*-value**
Age, years—median (IQR)	80 (74–85)	78 (72–84)	.006
Sex, male—*n/N* (%)	348/569 (61.2%)^a^	241/434 (55.5%)^b^	.073
NIHSS—median (IQR)	7 (3–13)^c^	6 (3–15)^d^	.978
mRS at 3 months follow-up—median (IQR)	6 (3–6)^e^	6 (3–6)^f^	.343
Mortality at 3 months—*n/N* (%)	246/488 (50.4%)^g^	182/324 (56.2%)^h^	.107
Onset-to-door time, minutes—median (IQR)	78 (51–227)^i^	94 (53–256)^j^	.373

Abbreviation: DNT = door-to-needle time; ICH = intracerebral haemorrhage.

Number of missing values: ^a^10; ^b^5; ^c^110; ^d^173; ^e^91; ^f^115; ^g^91; ^h^115; ^i^272; ^j^218.

ICH patients on anticoagulation with a known time of onset were younger (79 years vs 80 years, *P* < .001) and more often treated with a reversal agent than patients with a unknown time of onset (60.3% vs 51.8%, *P* < .001; Table 4). There were no differences in sex and NIHSS.

**Table 4:** Sub-analysis of ICH patients on anticoagulation, stratified by known vs unknown onset time.

	**Known time of onset (*n* = 941)**	**Unknown time of onset (*n* = 956)**	***P*-value**
Treated with reversal agent—*n/N* (%)	540/896 (60.3%)^a^	478/922 (51.8%)^b^	<.001
Age, years—median (IQR)	79 (73–84)	80 (75–86)	<.001
Sex, male—*n/N* (%)	503/925 (54.4%)^c^	518/940 (55.1%)^d^	.752
NIHSS—median (IQR)	9 (4–16)^e^	8 (3–17)^f^	.560

Abbreviation: ICH = intracerebral haemorrhage. Number of missing values: ^a^45; ^b^34; ^c^16; ^d^16; ^e^265; ^f^344.

## 3. Discussion

In this study, we compared the DNT between patients with ICH treated with a reversal agent and patients with AIS treated with IVT. We found that patients with ICH, on average, had a 21 min longer DNT than patients with AIS. After adjustment for baseline differences, DNT was still significantly longer for ICH patients. Clinical outcomes were also worse for ICH patients.

A national survey among Dutch neurologists showed that 56% estimated DNT to be longer for ICH than for AIS treated with IVT.^22^ Our findings support this perception, demonstrating that, despite the largely similar workflow for ICH and AIS patients regarding acute medical treatment, a clear difference in real-world treatment times exists.

Recent research in the United States reported similar findings to our study.^23^ Although this study compared time to anticoagulation reversal in ICH with endovascular treatment in AIS, as well as time to first antihypertensive in ICH with IVT in AIS, the overall conclusion remains unchanged: treatment for ICH is substantially slower than for AIS. This suggests that delayed ICH treatment is not limited to a single country but could represent a broader, potentially global issue.

Other research has demonstrated that a DNT of less than 60 min for anticoagulation reversal in ICH is associated with lower in-hospital mortality and a reduced likelihood of discharge to hospice care.^17^ In the present study, while the median DNT was below this 60-min threshold, this target was not achieved in a substantial proportion of patients.

Several limitations of our study should be considered when interpreting the results. First, due to the need to exclude a substantial proportion of patients because of missing DNT data in the ICH group, we cannot rule out the possibility of selection bias. Second, only limited information on relevant confounding patient characteristics and no detailed pre-hospital or extensive in-hospital data were available in the dataset. Lastly, there was a marked discrepancy in data completeness between ICH and AIS, with substantially more missing data in the ICH group, which may have reduced the reliability of findings for ICH patients and introduced potential bias.

To address missing data and their potential influence on our results, we performed a sensitivity analysis using multiple imputation, which showed a consistent difference in DNT, mRS and mortality between the ICH and AIS groups. Still, these results should be interpreted with care as the use of multiple imputation becomes less reliable with a higher proportion of missing data. To examine the possibility of selection bias, we compared baseline data of ICH patients with available DNT data to those without DNT. Apart from the patients with available DNT data being slightly older, no other significant differences were observed. The most plausible explanation for difference in proportion of missing DNT data between ICH and AIS patients is that, in 2023, variables related to reversal of anticoagulation in ICH were newly introduced, whereas treatment time for IVT is being collected for several years already. This may have resulted in the data collection for haemorrhage reversal not yet being as systematically embedded as the collection of IVT treatment times. Nonetheless, the marked difference in data completeness, DNT missing in only 4% of AIS vs 43% in ICH, may also suggest that, collectively, we have not yet attained the same level of rigor in capturing these data, even though this study once again shows that ICH patients have worse clinical outcomes.

A noteworthy finding of this research is that only 54% of ICH patients on anticoagulation were treated with a reversal agent. The data showed that patients with a unknown time of onset are less frequent treated with a reversal agent compared to patients with a known time of onset, 51.8% vs 60.3%. Reasoning behind this could be that the patients with a unknown time of onset were more likely to be past the most effective time window for anticoagulation reversal, and it was not considered to be effective anymore. In addition, other factors, such as haematoma volume, the patient’s pre-stroke condition and a decision on limitation of care may, have influenced the decision not to administer a reversal agent.

To better understand the reasons underlying the longer treatment time observed in patients with ICH compared to those with AIS, more detailed data on both pre-hospital and in-hospital processes are required. Logistical factors are likely to contribute, as the workflow for ICH management may be less optimized than that for AIS. The Dutch survey indicated for example that reversal medication is never administered in the CT suite and is not often delivered as a bolus.^22^ Intravenous thrombolytic agents such as alteplase and tenecteplase are readily available for IVT. In contrast, reversal agents such as prothrombin complex concentrate typically require several minutes to prepare and are often stored in the hospital pharmacy rather than in the emergency department. All these factors may contribute to procedural delays. The use of a tool that helps focus attention on timely management, such as the use of a structured checklist, could be valuable in optimizing the workflow for ICH patients.^24^ In addition, patient characteristics may also contribute to delayed treatment times. Patients with ICH more frequently present with a reduced consciousness and a compromised airway which may potentially delay multiple stages of care, including activation of emergency medical services, on-scene management and subsequent in-hospital care.^25^

In conclusion, treatment times for anticoagulation reversal in ICH patients are significantly longer than IVT for AIS patients in the Netherlands. Future research should aim to identify and characterize the specific sources of delay within the ICH workflow to enable targeted optimization, and should also further investigate other time-sensitive aspects of ICH management, including blood pressure-lowering strategies and the achievement of target blood pressure levels within recommended time frames. In parallel, it would be valuable for neurologists to assess, at the institutional level, which logistical components of their own local pathways could be improved to enhance overall efficiency of ICH management.

## Data Availability

Requests for the data can be submitted directly to DASA via onderzoek@dica.nl
